# Medical students with low self-efficacy bolstered by calling to medical speciality

**DOI:** 10.1007/s40037-014-0110-7

**Published:** 2014-02-15

**Authors:** Joel B. Goodin, Ryan D. Duffy, Nicole J. Borges, Catherine A. Ulman, Vanessa M. D’Brot, R. Stephen Manuel

**Affiliations:** 1 Department of Educational Psychology, Florida State University, 3295 Alice Drive, Batesville, AR 72501 USA; 2 University of Florida, Gainesville, FL USA; 3 Wright State University Boonshoft School of Medicine, Dayton, OH USA; 4 University of Cincinnati College of Medicine, Cincinnati, OH USA

**Keywords:** Calling, Medical students, Self-efficacy, Speciality choice, Medical education, Vocation, Career, Profession

## Abstract

This study was performed to understand the degree to which medical students’ self-efficacy (SE) moderates the influence of calling on students’ speciality commitment, emphasizing the need to understand variables that predict primary care specialization. The researchers hypothesized that students who perceived their career as a calling would be more committed to their speciality, especially when students had high SE. Medical students (Years 1–4; *N* = 152) completed an online survey to rate their calling, speciality commitment, and SE. Calling was measured by the Brief Calling scale (Dik et al., J Career Assess 20:242–263, 2012), while speciality choice was measured by Hollenbeck et al. (J Appl Psychol 74:18–23, 1989) measure of commitment. SE was measured by the Jerusalem and Schwarzer's general SE scale (see Scholz et al., Eur J Psychol Assess 18:242–51, 2002). Calling (*r* = 0.24, *p* < 0.01) and SE (*r* = 0.20, *p* < 0.05) were found to moderately correlate with speciality commitment, thus emphasizing the possibility that they may have an interaction. The interaction of calling and SE significantly predicted speciality commitment (*β* = −0.20, *t*(148) = −2.55, *p* < 0.05) and explained a significant proportion of variance in speciality commitment (*R* ^2^ = 0.12, *F*(3, 148) = 6.875, *p* < 0.001). Students with a high presence of calling may have high speciality commitment, despite low SE.

## Introduction

Making a commitment to a particular speciality is a critical benchmark in a medical student’s career development. The United States currently has a great need for generalist physicians in the wake of the ongoing healthcare reform. Thus, it is important to understand what factors predict students feeling confident and decided in their speciality choice. Despite decades of literature on speciality choice influences [1], medical education researchers continue to investigate the ways in which students commit to specific specialities. Our study adds to the literature by investigating career calling, the ‘feeling that one’s career is a calling,’ as one potential predictor of speciality commitment. Career calling is hypothesized to relate to commitment, especially for students at different levels of confidence in their ability to perform in medical school (i.e., self-efficacy, SE).

### Committing to a speciality

Medical students have great freedom in choosing a speciality for which they are interested and capable. With over 125 medical specialities from which to choose, students base their speciality choices on fiscal and social gains as well as variables such as intrinsic interest, aptitude, and opportunity, among a vast array of decision criteria [1]. Applying for a certain speciality, such as neurosurgery or plastic surgery, may involve greater competition due to less availability of residency positions and the potential for higher salaries and greater prestige [2].

Medical students must be confident in medical school tasks, such as displaying the capability to outperform the competition in their intended speciality to secure their preferred residency placement [2]. Bandura [3] was the first to use the term SE to indicate one’s confidence toward specific, future goals. A medical student would be more likely to have high SE in obtaining residency placement in the speciality of their choice if they outperform their classmates in coursework and on the United States Medical Licensure Exam: One (USMLE Step 1). A medical student who has shown a lesser degree of capability would likely have lower SE regarding their speciality of choice such that they might modify their commitment to a more attainable speciality option.

### Calling among medical students

A calling to a career choice has been emphasized as a relevant consideration among undergraduate students and working adults [4]. Several recent studies have examined how calling functions among a medical student population. In 2013, Borges et al. [5] found that first-year medical students reported moderate to high levels of calling toward a speciality. Furthermore, medical students interested in generalist specialities were more likely to espouse a calling than those who were interested in non-generalist specialities. Duffy et al. [6] noted the decrease in perception of medical students’ career as a calling after the first 2 years. Based on findings [7, 8] that have associated calling with commitment, speciality choice was operationalized in the current study as commitment to one’s most likely medical speciality choice.

### SE as a moderator

Scholars who study the concept of a calling have noted that feeling a calling may be especially important for individuals in highly challenging jobs and/or individuals who face added barriers to deciding on a particular career [9]. For these individuals, calling is hypothesized to function as a protective mechanism which supports motivation and decision-making when facing barriers to career commitment. One notable barrier to commitment for medical students may be insufficient SE in their medical career goals. For students with lower levels of SE, it may be especially important to feel a calling in order to build commitment to a specific speciality.

Empirical research has supported the relationship of calling and commitment. More importantly, recent research among a variety of populations has consistently found that a students’ career calling was positively related to their career SE [10–12]. The association between calling and SE presents the possibility of an interactive effect on speciality commitment which is hypothesized in this study.

### The present study

The goal of the present study is to examine the link between presence of calling and general SE (GSE) to speciality commitment among medical students. Our research questions ask ‘What is the relationship between medical students’ commitment to a speciality choice and their perception of a calling to their career?’ and ‘What role does SE play in the relationship of medical students’ speciality choice commitments and their calling to their career?’ Based on previous findings [13], the researchers hypothesize that students who view their career as a calling will be more committed to their speciality choice. Additionally, based on the potential bolstering effect of calling in the face of challenges (i.e., decreased SE), we hypothesize that the link between calling and speciality commitment will be most pronounced with students who have lower levels of SE.

## Method

### Procedure

During the fall of 2012, with institutional review board approval, medical students (*N* = 1,060) enrolled in Years 1–4 at two Midwestern United States medical schools were e-mailed an invitation to participate in the study, along with a link to surveys which measured the presence of a calling, speciality commitment, and SE. Postgraduate students (i.e., Year 5 and beyond) were excluded in order to focus on students in graduate medical training.

Completion of the survey was voluntary. While 105 of a potential 400 participants at Medical School ‘A’ chose to participate (26.3 %), 97 of 660 potential participants at Medical School ‘B’ chose to participate (14.7 %). The 50 % female representation in the final sample was not dissimilar to the gender distribution in the population. Depending on their medical school, students were e-mailed either one or two reminders before closure of the online survey and may have been invited to enter a raffle to win one of five $100 Visa gift cards as incentive for their participation. The survey was part of a larger data collection related to a project on health care reform that formed the basis for a dissertation of the current study’s first author.

### Instruments

#### Presence of calling

The Presence subscale of the Brief Calling scale (BCS) was used to assess the degree to which students perceived a calling in their career [14]. Items were answered on a five-point Likert-type scale ranging from (1) *not at all true of me* to (5) *totally true of me*, and scores on the two items were added for a total presence of calling score. In the validation study, Dik et al. [14] found BCS scores to correlate positively with scores on other measures of calling and with informant reports of participants’ perceptions of calling. Additionally, BCS scores have been correlated with aspects of career maturity and well-being in previous studies [4, 13]. In the current study, SPSS was used for all statistical analyses. While degree of reliability (low, moderate, or high) is somewhat subjective, the researchers regarded absolute values of 0.4–0.59 as low, 0.6–0.8 as moderate, and greater than 0.8 as high. A Spearman’s *ρ* coefficient measure of the internal consistency of the two-item scale showed high reliability (*ρ* = 0.80). Spearman’s *ρ* was used for this scale in lieu of Cronbach’s *α* to measure its reliability because the scale had only two items.

#### Speciality commitment

In order to measure students’ commitment to medical goals, the three highest loading general commitment items were adapted from Hollenbeck et al.’s (HWK) goal commitment scale for medical students. Specifically, item phrasing was modified such that ‘this goal’ was replaced with ‘this speciality’ [15]. The HWK is a self-report scale which is the most commonly used measure of goal commitment in extant research literature [16]. For the current study, the HWK was used to measure medical students’ commitment to their ‘most likely’ speciality. An example of an item from the instrument is ‘I am absolutely committed to pursuing this speciality.’ Items were answered on a six-point Likert-type scale ranging from (1) *strongly disagree* to (6) *strongly agree*, and scores on the three items were added for a total speciality commitment score. In the current study, the scale showed high reliability using Cronbach’s *α* coefficient of internal consistency (*α* = 0.88).

#### General SE

Medical students’ SE beliefs were measured by the GSE scale, an instrument developed by Jerusalem and Schwarzer [17]. The measure was intended to ‘assess a general sense of perceived SE with the aim in mind to predict coping with daily hassles as well as adaptation after experiencing all kinds of stressful life events’ (Scholz et al. [17], p. 29).

High reliability, stability, construct validity, and criterion validity of the GSE scale has been supported by many studies (e.g., Luszczynska et al. [18]) and with many populations (e.g., 23 nations). The GSE scale has an average Cronbach’s *α* of 0.88 [17]. Luszczynska et al. [18] reviewed multiple studies that found GSE scores to be positively related to job satisfaction, positive emotions, and optimism, and negatively related to stress, burnout, health complaints, depression, and anxiety.

All 10 of the original GSE items were answered on a six-point Likert-type scale ranging from (1) *strongly disagree* to (6) *strongly agree*, and scores on the 10 items were added for a total SE score. In the current study, the scale showed high reliability using Cronbach’s *α* coefficient measure of internal consistency (*α* = 0.94).

## Results

A total of 202 medical students completed the survey with a 20.5 % response rate. Participants spanned Years 1–4 of medical school while some reported ‘Other’ as their year (e.g., dual degree students). The sample included students ranging in age from 21 to 38 years with a mean age of 23.43 (SD = 0.82). With regard to the participants’ gender, males and females were closely represented in the sample. Four primary ethnographic groups were represented in the sample, with similar representation across the two schools (Table 1).

**Table 1:** Participating medical school characteristics

Demographic variables	School A (%)	School B (%)	Total (%)
Overall response rate	105 (26.3 %)	97 (14.7 %)	202 (20.5 %)
Year			
Year 1	25 (23.8 %)	0 (0.0 %)	25 (12.4 %)
Year 2	33 (31.4 %)	37 (38.1 %)	70 (34.7 %)
Year 3	18 (17.1 %)	18 (18.6 %)	36 (17.8 %)
Year 4	24 (22.9 %)	38 (39.2 %)	62 (30.7 %)
Year (other)	5 (4.8 %)	4 (4.1 %)	9 (4.5 %)
Age			
Age 21–22	11 (10.5 %)	1 (1.0 %)	12 (5.9 %)
Age 23–25	65 (61.9 %)	54 (55.7 %)	119 (58.9 %)
Age 26–28	19 (18.1 %)	28 (28.9 %)	47 (23.3 %)
Age 29–32	8 (7.6 %)	13 (13.4 %)	21 (10.4 %)
Age 33 and older	2 (1.9 %)	1 (1.0 %)	3 (1.5 %)
Gender			
Male	50 (47.6 %)	52 (53.6 %)	102 (50.5 %)
Female	55 (52.4 %)	45 (46.4 %)	100 (49.5 %)
Ethnicity			
African American	5 (4.8 %)	5 (5.2 %)	10 (5.0 %)
Asian	10 (9.5 %)	15 (15.5 %)	25 (12.4 %)
Caucasian	86 (81.9 %)	74 (76.3 %)	160 (79.2 %)
Hispanic or Latino	3 (2.9 %)	1 (1.0 %)	4 (2.0 %)
Missing	1 (1.0 %)	2 (2.1 %)	3 (1.5 %)

*N* = 202

First, we examined the simple bivariate correlation between calling, SE, and speciality commitment. Average answers to each questionnaire item can be seen in Table 2.

**Table 2:** Mean answers to questionnaire items

Questionnaire items	Mean scores	SD
Presence of calling		
Item 1	3.51	1.21
Item 2	3.42	1.14
Self-efficacy		
Item 1	4.72	1.10
Item 2	3.91	1.12
Item 3	4.75	0.98
Item 4	4.86	0.95
Item 5	4.91	0.95
Item 6	5.10	0.91
Item 7	5.10	0.89
Item 8	4.95	0.90
Item 9	5.04	0.83
Item 10	5.00	0.87
Speciality commitment		
Item 1	5.11	1.04
Item 2	5.06	0.87
Item 3	4.88	1.17

*N* = 202

**Fig. 1:**
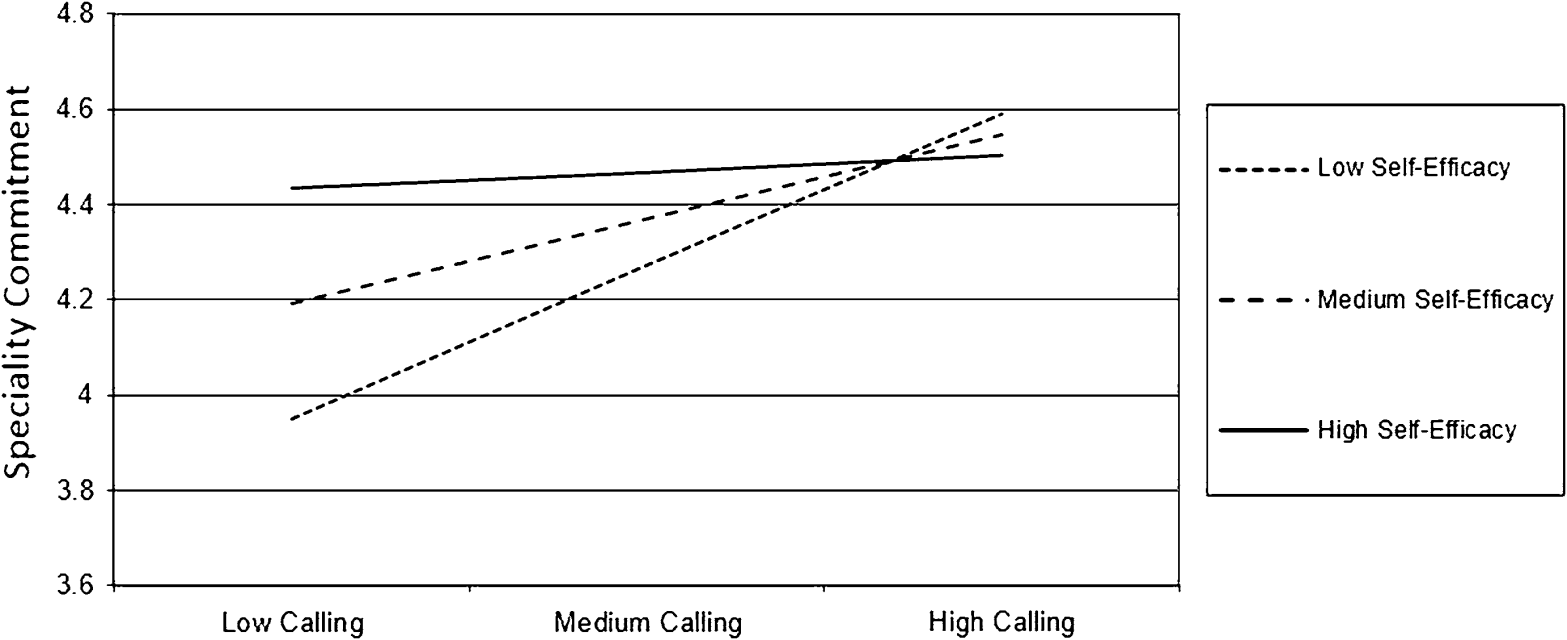
Self-efficacy as a moderator in the relation of calling and speciality commitment

Calling (presence of calling, *r* = 0.28, *p* < 0.001) and SE (*r* = 0.20, *p* = 0.004) were found to moderately correlate with speciality commitment. There were no noteworthy demographic differences on calling, SE, or speciality commitment except that males were on average more self-efficacious than females (*M* = 4.98 and 4.68, respectively). Second, we examined the degree to which SE moderated the relation between calling and speciality commitment. As seen in Fig. 1, SE is depicted with three lines representing low, mean, and high levels of SE (true range 1.1–6.0) corresponding with values 1 SD below the mean (4.07), at the mean (4.86), and 1 SD above the mean (5.65). Analogously, three levels of calling (range 1–5) are also depicted with values 1 SD below the mean (2.57), at the mean (3.58), and 1 SD above the mean (4.59). These are plotted with speciality commitment as the dependent variable (range 2–6; Table 3).

**Table 3:** Self-efficacy as a moderator in the relation of calling and speciality commitment

	*β*	*B*	SE *B*	95 % CI	*R*	*R* ^2^	*R* ^2^ *Δ*	*FΔ*
Step 1								
Calling	0.25***	0.20	0.06	0.09 to 0.32				
Self-efficacy	0.15*	0.19	0.08	0.02 to 0.35	0.31	0.10		
Step 2								
Calling × self-efficacy	−1.2**	−0.18	0.06	−0.30 to −0.05	0.36	0.13	0.03	7.50

* *p* < 0.05, ** *p* < 0.01, *** *p* < 0.001

As depicted in Fig. 1, the relation of calling to speciality commitment was stronger for students at lower levels of SE. Although not among our original hypotheses, but guided by the work of Borges et al. [5], the relationship of one’s presence of calling to their interest in generalist versus non-generalist careers was also explored. Interest in the two categories of speciality was measured by having medical students rate their interest in generalist and non-generalist specialities on a 10-point Likert-type scale ranging from (1) *no commitment* to (10) *absolutely committed*. Presence of calling was more prevalent among those more interested in generalist careers, such that presence of calling scores explained a significant amount of variance in generalist interest ratings, *F*(8, 193) = 2.174, *p* < 0.05.

### Additional findings

In order to better understand the interaction of SE and calling, the relationships of speciality commitment, SE, and calling with year were each analyzed. Though not a hypothesis of the current study, the year of the medical student was assumed to be positively related to speciality commitment but not to calling or SE based on previous research [19]. Subsamples by year did not differ with regard to calling or SE, but did significantly differ with regard to speciality commitment, *F*(4, 197) = 14.541, *p* < 0.001.

With the hypothesis that presence of calling would be greater for students who were strongly committed to less prestigious and fiscally rewarding medical specialities, the researchers sought to identify across and between schools’ specialities that were associated with high ‘presence of calling’ scores (i.e., mean score of 3.5 or above out of 5). In both schools, high calling scores were most often associated with generalist specialities (internal medicine, 20 individuals; paediatrics, 10; family medicine, 7), although School A students reported comparatively greater commitment to generalist specialities in some areas such as paediatrics (8 individuals).

Philosophical differences add perspective to differences between the schools regarding calling to specific specialities [20]. School A consistently graduates more generalist physicians than other medical schools in its geographic area and is rated among the top five United States medical schools in its dedication to a ‘social mission,’ as assessed by a compilation of the generalist physicians produced, work by graduates in Health Professional Shortage Areas and minority student representation. While School A has a focus on generalist training, School B has a broader focus that encompasses a wider range of clinical and scientific training. With regard to this focal difference, School A is a community-based, or ambulatory, medical school while School B uses the more traditional university-based hospital for training. Community-based training has been associated with an approximate 9 % increase in generalist specialization among students who encounter it [21, 22].

## Discussion

The choice of a medical student’s speciality results from a complex set of factors. Financial compensation, social and familial expectations, medical school experiences, and personal factors have often been noted as major influences on students’ speciality choices (e.g., Yang and Tsai [23]) More recently, Chang et al.’s [24] research noted speciality characteristics and the speciality training process as highly influential. Females were found to place more emphasis on future lifestyle.

The current study explored the potential influence that the psychological variable, SE, and the presence of calling to a career might have on medical students’ speciality choices. Consistent with the findings of Goodin [19], students with higher GSE tend to be more committed to a specific speciality. It may be that students who are generally more self-efficacious display a deeper loyalty to the medical speciality to which they aspire. However, due to the correlational nature of the study, there remains the possibility that those who have found a speciality that is a ‘good fit’ will have high SE. For example, students who know they have the intelligence and skill to aspire toward a speciality are likely to report an increase in their confidence (i.e., SE) to perform the tasks that their speciality, career, and life require. It is noteworthy that there was no gender difference among participants for calling, speciality commitment, or the interaction of calling and SE, although males were on average more self-efficacious than females.

In contrast to the skill-to-trade match that often influences career choice, the current study sought to understand the degree to which a student’s calling to the medical career was explanatory of their commitment to their speciality choices. Findings of the current study indicate that medical students who report a high degree of calling toward their career are also highly committed to their speciality choice. This finding lends support to assertions of Senf et al. [25] that the generalist workforce may be increased the most by enrolling students already interested, or perhaps ‘called,’ to generalist specialities. In contrast, medical students reporting a low degree of calling to their career had much lower degrees of commitment to their speciality choice when SE was not considered (i.e., controlled). However, the study showed that the degree of SE of the medical student moderated the relationship of medical student calling and speciality commitment, such that when SE ratings were high, a medical student reported a high degree of speciality commitment regardless of their calling ratings. It is reasonable to interpret the role of SE as a bolster to the influence of calling on speciality commitment. Simply put, students who may feel ‘called’ to a specific speciality may still be highly committed to that speciality if they have the repertoire of skills and intelligence that supports success in that speciality. When a student’s calling is insufficient to inspire commitment, the rational match of the student’s abilities to the task increases SE and, consequently, speciality commitment.

While calling may, perhaps, originate and inspire an individual as early as grade school, the commitment to a speciality choice and a final decision often develops the most during medical school [19]. Students often have an idea of what they hope to specialize in as they enter medical school, but their choice often changes multiple times before they make a more official decision in their third year of medical school as they start to apply for residency positions. It should be noted, however, that many students continue to change their minds during their fourth year and postgraduate years. The current study did not include hypotheses regarding the relationship of calling or speciality commitment to students’ years in medical school. However, since subsamples by year did not differ with regard to calling or SE, there was no evidence that year played a confounding role in the identified interaction. The lack of difference in calling by year is inconsistent with previous research by Duffy et al. [6].

Finally, it is noteworthy that the relationship of presence to generalist speciality interest [5] was replicated in the current study. It is commonly known that generalist specialities garner less financial and social reward (e.g. prestige). Therefore, it may be that those who are called (i.e., intrinsically motivated) to serve as generalist physicians may be more likely to remain committed to generalist specialities that carry less extrinsic rewards. Organismic Integration Theory [26], which delineates a continuum of intrinsic and extrinsic motivation, may be an appropriate theoretical basis on which to temporarily frame calling research and literature until a more specific calling theory is developed.

### Limitations and implications

In light of published research (e.g., Paolo et al. [27]) and medical school ‘in-house’ response rates shared by school administrators, the sample’s response rate was expected to be as low as 10–20 % for medical students taking an online survey. Two primary ad hoc strategies were employed to improve the response rate: incentives (i.e., raffle opportunity) and two survey reminders. The school without the incentive sent two reminders, while the school with the incentive opportunity sent only one reminder. The techniques could only be employed to the extent allowed by the medical school administration and human subjects purview. Despite the differences in methodology, both schools had equivalent numbers of participants, though response rates were somewhat lower in the school that did not allow students to participate in a raffle (26 vs. 15 %). According to Charlton [28], there is no official acceptable response rate, although a 33 % response rate is common for online surveys [29]. Medical student response rates for online surveys have been found to be somewhat lower than other populations [27]. The average response rate of 21 % for the current sample is understandable in light of the administrative modifications to the methodology for both schools and the heavy daily work schedule of medical students, generally, and specifically for the students at these specific schools during the time of survey administration explained by administrators from both schools.

Caucasian students were overrepresented in the study, providing a less diverse sample that limits the generalizability of the findings to a national population of medical students. The Association of American Medical Colleges [30] reported a 59 % representation of Caucasians in their national sample. The similar geographic location of both schools (i.e., Midwestern United States) may have contributed to homogeneity of students across both schools. Future studies of medical students’ calling, SE, and speciality commitment should include a larger, more heterogeneous sample to provide more generalizable findings.

In conclusion, students more likely to view their career as a calling are more committed to their speciality choice. For students with lower levels of SE, it may be especially important to feel a calling in order to build commitment to a specific speciality. This study’s findings have implications for counselling and advising medical students. Calling is an under-incorporated variable in counselling and advising students on medical speciality choice. Exploring a student’s calling and its relation to speciality commitment may be helpful to students who have lower levels of SE and/or students who are struggling with choosing a medical speciality. Determining ways to increase a student’s calling to medicine is an important next step to further this line of inquiry.

## Essentials

- Presence of calling and SE beliefs were significantly related to speciality commitment.
- Presence of calling was more prevalent among those more interested in generalist specialities.
- The interaction of calling and SE significantly predicted speciality commitment, explaining a significant proportion of speciality commitment variance.
- Students with a high presence of calling may have high speciality commitment, despite low SE.
- Exploring a student’s calling and its relation to speciality commitment may be helpful to students who have lower levels of SE and/or students who are struggling with choosing a medical speciality.
